# Collaborative assessment of the risk of postoperative progression in early-stage non-small cell lung cancer: a robust federated learning model

**DOI:** 10.1186/s40644-025-00911-y

**Published:** 2025-07-18

**Authors:** Yu Liu, Xiaobei Duan, Xiaojuan Chen, Kunwei Li, Qiong Li, Ke Liu, Wansheng Long, Huan Lin, Bao Feng, Xiangmeng Chen

**Affiliations:** 1 Chongqing Big Data Collaborative Innovation Center, Chongqing, China; 2 Laboratory of Intelligent Detection and Information Processing, Guilin University of Aerospace Technology, Guilin, China; 3 Department of Nuclear Medicine, Jiangmen Central Hospital, Jiangmen, China; 4 Department of Radiology, Jiangmen Central Hospital, Jiangmen, China; 5 Department of Radiology, The Fifth Affiliated Hospital of Sun Yat-sen University, Zhuhai, China; 6 Department of Radiology, Sun Yat-sen University Cancer Center, Guangzhou, China; 7 School of Computer Science and Technology, Chongqing University of Posts and Telecommunications, Chongqing, China; 8 Jiangmen Key Laboratory of Artificial Intelligence in Medical Image Computation and Application, Jiangmen Central Hospital, Jiangmen, China; 9 Department of Radiology, Guangdong Provincial People’s Hospitial, Guangzhou, China

**Keywords:** Non-small cell lung cancer, Postoperative progression, Federated learning, Multicenter study

## Abstract

**Background:**

While the TNM staging system provides valuable insights into the extent of disease, predicting postoperative progression in early-stage non-small cell lung cancer (NSCLC) remains a significant challenge. An effective bioimaging prognostic marker for early-stage NSCLC, powered by artificial intelligence, could greatly assist clinicians in making informed treatment decisions.

**Methods:**

A total of 926 patients from four centers (A, B, C, and D) with histologically confirmed stage I or II solid non-small cell lung cancer (NSCLC) who underwent surgical resection were retrospectively reviewed. In this study, we propose a robust federated learning model (RFed) designed to predict the risk of postoperative progression in early-stage NSCLC patients. The diagnostic efficiency of the RFed model was evaluated using the area under the curve (AUC) and Decision Curve Analysis (DCA). Additionally, the model’s performance was further validated through Kaplan-Meier survival analysis, with statistical significance assessed using the log-rank test. Finally, the robustness, generalizability, and interpretability of the RFed model were comprehensively evaluated to confirm its clinical applicability.

**Results:**

Experimental results demonstrated the superior performance of the RFed model. Specifically, RFed achieved AUC values of 0.936, 0.861, 0.925, and 0.970 on the test sets from the four centers. DCA further revealed that RFed provided a greater net benefit compared to the clinical model across a threshold probability range of 0.02 to 0.99. Moreover, Kaplan-Meier curves showed improved discrimination between high-risk and low-risk groups when compared to other models, highlighting its enhanced predictive capability.

**Conclusions:**

The RFed model demonstrates significant effectiveness in predicting the risk of postoperative progression in early-stage NSCLC patients. Its clinical application value lies in its potential to enhance stratified management and support the development of precise treatment strategies for this patient population.

**Supplementary Information:**

The online version contains supplementary material available at 10.1186/s40644-025-00911-y.

## Introduction

Lung cancer represents a leading cause of malignancy, with non-small cell lung cancer (NSCLC) being the most prevalent subtype, accounting for approximately 85% of all cases [1, 2]. According to the National Comprehensive Cancer Network (NCCN) guidelines, anatomic lung resection and lymph node dissection are the primary surgical treatments for patients with NSCLC [3]. Despite these interventions, the rate of postoperative progression, including recurrence and metastasis, remains high, with reported rates ranging from 18 to 34% [4]. Currently, the tumor node metastasis classification (TNM) system is widely used for prognostic assessment in lung cancer patients. Unfortunately, the clinical manifestations and prognostic factors of NSCLC exhibit significant heterogeneity, leading to different outcomes even among patients with the same TNM stage [5]. Therefore, there is an urgent need to develop an accurate method capable of identifying high-risk patients prone to postoperative progression, thereby enabling early intervention (such as adjuvant chemotherapy and close follow-up) and improving patient outcomes.

In recent years, deep learning (DL) has gained considerable attention in the medical field, achieving remarkable success in diverse applications, including lung cancer prognosis [6], bladder cancer treatment [7], and preoperative grading in meningioma [8]. The development of robust and reliable DL-assisted diagnostic systems relies not only on advanced algorithms but also on the valuable knowledge extracted from large-scale datasets collected across multiple institutions [9]. However, constructing ever-larger centralized datasets is unsustainable for smart healthcare systems due to concerns over patient privacy and ethical issues.

Federated Learning (FL) [10], a distributed machine learning framework, enables multiple data owners to conduct local model training and aggregate models on a central server iteratively. Without sharing raw data, the globally aggregated model can outperform models trained on individual centers. Among existing FL approaches, personalized FL [11] is particularly suitable for medical scenarios, allowing each center to choose between the aggregated global model and local models based on their needs. However, real-world medical FL faces challenges such as data heterogeneity caused by differences in imaging devices, protocols, and regional disease characteristics. Conventional model parameter aggregation strategies can lead to performance degradation in local models after each communication. Additionally, class imbalance in medical imaging, such as postoperative progression occurring in only around 20% of NSCLC patients [12], can further hinder model performance.

In this study, we propose a robust federated learning model (RFed) designed to predict the risk of postoperative progression in early-stage NSCLC patients. Our model aims to support more informed and personalized clinical decisions, ultimately improving patient outcomes in early-stage NSCLC.

## Materials and methods

### Data acquisition and pre-processing

The study retrospectively analyzed clinical and radiological data from 926 patients across four centers who were diagnosed with solid NSCLC, underwent surgical resection, and received pathological confirmation between January 2014 and September 2019. The inclusion criteria were as follows: (1) Diagnosis of solid NSCLC confirmed by pathological examination following surgical resection; (2) Chest Computed tomography (CT) examination performed within one month prior to surgery; (3) Availability of CT images from the Picture Archiving and Communication System (PACS); (4) Regular and complete follow-up records for at least three years after surgery; (5) Pathological staging in stages I or II; (6) Image slice thickness ≤ 1.5 mm. The exclusion criteria were as follows: (1) Patients with concurrent malignancies; (2) Patients without postoperative follow-up or with a follow-up duration of less than three years; (3) Pathological staging in stages III or IV. (4)Lung cancer lesions manifested as sub-solid nodules (both of non-solid and part-solid). (5)Consolidation lesions with indistinct margins (pneumonic-type lung adenocarcinoma) and central lesions complicated with obstructive atelectasis.

The data from these four centers were randomly divided, with patients allocated to four groups for training, validation, and testing, as shown in Table 1.

**Table 1:** Basic information about the patients included in this study

Center	Data Set	Disease type LCP vs. LCNP	Age, year (Mean ± SD)	Gender		longest diameter, cm (Mean ± SD)	Lobulated		Spiculated		Smoking history		CEA	
				male	female		absent	present	absent	present	absent	present	normal	elevated
A (*n* = 198)	Train (*n* = 96)	LCP(*n* = 20)	62.90 ± 11.79	14	6	3.50 ± 1.83	1	19	4	16	12	8	12	8
		LCNP(*n* = 76)	59.07 ± 10.48	44	32	2.79 ± 1.47	7	69	40	36	54	22	62	14
	Validation (*n* = 24)	LCP(*n* = 5)	63.20 ± 10.99	4	1	2.73 ± 1.03	0	5	2	3	1	4	1	4
		LCNP(*n* = 19)	60.26 ± 9.27	12	7	2.22 ± 1.39	3	16	10	9	16	3	14	5
	Test (*n* = 78)	LCP(*n* = 14)	69.29 ± 7.29	8	6	2.59 ± 0.91	3	11	3	11	10	4	6	8
		LCNP(*n* = 64)	63.48 ± 10.01	35	29	2.37 ± 1.20	7	57	34	30	46	18	54	10
B (*n* = 465)	Train (*n* = 224)	LCP(*n* = 40)	61.05 ± 10.27	23	17	2.91 ± 1.78	5	35	16	24	26	14	24	16
		LCNP(*n* = 184)	60.15 ± 10.75	97	87	2.30 ± 1.75	29	155	105	79	132	52	151	33
	Validation (*n* = 56)	LCP(*n* = 10)	61.60 ± 7.06	7	3	3.17 ± 1.40	1	9	3	7	8	2	8	2
		LCNP(*n* = 46)	60.22 ± 11.18	24	22	2.31 ± 1.70	7	39	24	22	36	10	36	10
	Test (*n* = 185)	LCP(*n* = 32)	62.09 ± 10.29	21	11	3.64 ± 1.93	5	27	13	19	25	7	22	10
		LCNP(*n* = 153)	60.28 ± 9.45	82	71	2.53 ± 1.85	27	126	78	75	103	50	120	33
C (*n* = 148)	Train (*n* = 72)	LCP(*n* = 12)	58.17 ± 13.33	7	5	3.17 ± 2.44	1	11	10	2	7	5	9	3
		LCNP(*n* = 60)	59.32 ± 9.99	31	29	2.26 ± 2.41	13	47	40	20	36	24	44	16
	Validation (*n* = 18)	LCP(*n* = 3)	53.67 ± 15.37	1	2	2.76 ± 2.98	1	2	2	1	2	1	3	0
		LCNP(*n* = 15)	60.60 ± 11.40	9	6	2.48 ± 1.65	5	10	9	6	9	6	11	4
	Test (*n* = 58)	LCP(*n* = 11)	57.45 ± 9.31	7	4	2.78 ± 1.71	2	9	5	6	5	6	8	3
		LCNP(*n* = 47)	56.02 ± 9.68	25	22	2.21 ± 1.77	10	37	31	16	27	20	43	4
D (*n* = 115)	Train (*n* = 56)	LCP(*n* = 8)	61.13 ± 5.87	5	3	2.01 ± 0.35	0	8	0	8	7	1	6	2
		LCNP(*n* = 48)	57.92 ± 11.21	28	20	2.12 ± 0.35	0	48	0	48	36	12	36	12
	Validation (*n* = 14)	LCP(*n* = 2)	60.50 ± 2.12	1	1	1.90 ± 1.28	0	2	0	2	2	0	2	0
		LCNP(*n* = 12)	65.00 ± 0.90	5	7	2.01 ± 0.45	0	12	0	12	6	6	7	5
	Test (*n* = 45)	LCP(*n* = 7)	57.86 ± 7.36	3	4	2.36 ± 0.41	0	7	0	7	3	4	5	2
		LCNP(*n* = 38)	58.89 ± 11.36	18	20	1.88 ± 0.44	0	38	0	38	25	13	31	7

Notes: LCP: lung cancer progression. LCNP: lung cancer non-progression. CEA: carcinoma embryonic antigen. SD: standard deviation

In order to accommodate the inputs of the deep learning model and to fully utilize its automatic feature extraction capabilities, this study used a bounding box for coarse segmentation of the region of interest (ROI) and did not use pixel-level labeling. Of course, to be precise, the All lesion was initially labeled with a bounding box by radiologist with over 10 years of experience, and then fully reviewed and validated by a second independent radiologist. This dual review process focuses on confirming the complete inclusion and correct identification of the lesion within the box, prioritizing localization suitable for deep learning analysis over fine-grained boundaries. To fit the input to the neural network, all segmented lesion images were subsequently normalized to a size of 128 × 128 pixels. A detailed description of the CT scanning parameters and the data preprocessing process is provided in Supplementary S1.

### Follow-up record and definition of postoperative progression

Follow-up: (1) Chest CT scans are performed every 6–12 months during the first two years post-surgery, and annually thereafter. (2) If clinical symptoms arise, site-specific examinations are conducted as needed. (3) The study endpoint is defined by the occurrence of disease progression. Patients without progression will be followed for a minimum of three years.

The definition of progression is as follows: (1) Postoperative follow-up progression is assessed through a combination of physical examinations, imaging, and tumor marker testing [13]; (2) Local progression is identified by the appearance of lesions at the surgical margins, within the same side of the thoracic cavity, or in the mediastinum; (3) Distant metastasis refers to lesions appearing in organs outside the contralateral lung, thoracic cavity, and mediastinum [14]; (4) When clinically feasible, a definitive diagnosis is confirmed through pathological histology or cytology examinations [14]; (5) Suspected recurrent or metastatic lesions that lack histopathological confirmation but show size increase during follow-up, or decrease in size after tumor-suppressive treatment, are clinically diagnosed as recurrence or metastasis [13].

Specifically, patients who experience progression within three years are classified into the positive group, while those without progression in the same period are placed in the negative group.

### Construction of a prognostic model based on robust federated learning

**Fig. 1:**
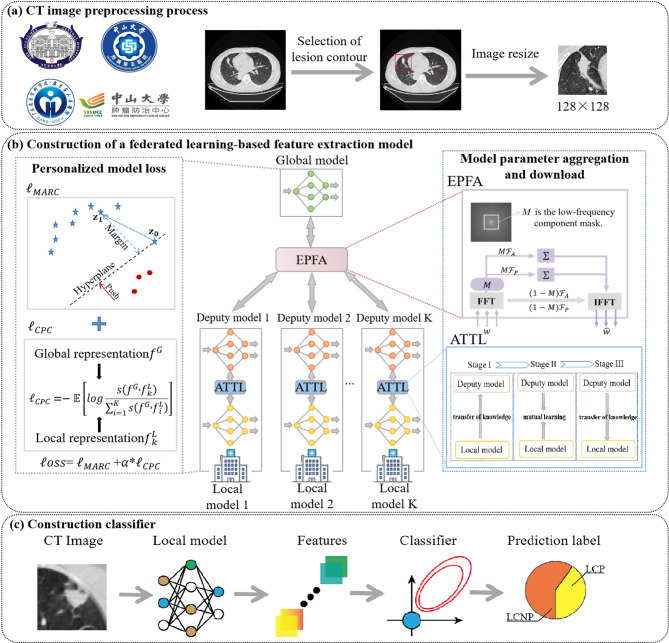
The flowchart of the overall study design. (**a**) CT image preprocessing process. (**b**) Construction of a federated learning-based feature extraction model. (**c**) Construction of a classifier using a Bayesian extreme learning machine

Traditional parameter aggregation and downloading strategy typically involve directly averaging the model parameters from multiple centers before transmitting them back. However, since parameters from different center models may represent distinct semantic patterns at the same position, direct averaging the model parameters can undermine the personalization of the models. This issue is particularly critical in early-stage NSCLC diagnosis, where multicenter data exhibit both heterogeneity and class imbalance. In such cases, directly applying global model parameters to each local model may lead to performance degradation. To address these challenges, this study proposes a robust federated learning (RFed) algorithm designed for handling multicenter, imbalanced, and heterogeneous medical images, as illustrated in Fig. 1. The RFed algorithm consists of two main components: (1) construction of a federated learning-based feature extraction model, and (2) construction of a classifier using a Bayesian extreme learning machine.

**(1) Construction of feature extraction model**: The construction of feature extraction model introduced two key improvements: ①the model parameter aggregation and download strategy, and ②personalized model loss for handling class imbalance in samples.

①**The model parameter aggregation and download strategy**.

For the model parameter aggregation and download strategy, this study employs a partially personalized Exponential Progressive Fourier Aggregation (EPFA) strategy, where low-frequency model parameters are aggregated and shared through Fourier Transform, while high-frequency parameters remain localized. This approach helps mitigate performance degradation that may arise from full model parameter aggregation in the time domain [15]. The pseudo-code of EPFA as shown in Algorithm 1. Here, $\:G$ is the global model, $\:D$ is the deputy model, $\:T$ is the number of communication, $\:M$ is the low-frequency mask matrix, $\:K$ is the number of clients, and $\:{\text{r}}_{\text{m}\text{a}\text{x}}$ is the threshold for the low-frequency components.

**Table:** 

Algorithm 1 Exponential Progressive Fourier Aggregation (EPFA)	
1: Initialize Global model $\:G$, deputy model $\:D$, $\:{\text{r}}_{\text{m}\text{a}\text{x}}$, $\:T$, $\:K$ 2: for each communication round $\:t=0$ to $\:T-1$ do 3: // **Client-side processing** 4: for each client $\:k=1$ to $\:K$ in parallel do 5: $\:{\theta\:}_{k}=LocalUpdate\left(D\right)$ 6: $\:{A}_{k},\:{P}_{k}=FFT\left(Reshape\right({\theta\:}_{k}\left)\right)$ 7: end for 8: // **Server aggregation** 9: $\:{A}_{avg}=\frac{1}{K}{\sum\:}_{k}{A}_{k}$, $\:{P}_{avg}=\frac{1}{K}{\sum\:}_{k}{P}_{k}$ // Amplitude and phase aggregation 10: ${F_{avg}} = {A_{avg}} \odot exp(j \cdot {P_{avg}})$ // Reconstructing frequency domain parameters 11: $\:\:{r=r}_{max}*{(T-t)}^{-1}$ // Low frequency threshold update 12: $\:M=LowFreqMask\left(r\right)$ // Low-frequency mask matrix 13: ${F_{low}} = {F_{avg}} \odot M$ // Extraction of low frequency components (high frequency zero setting) 14: $\:{\theta\:}_{aggregated}=IFFT\left({Reshape}^{-1}\right({F}_{low}\left)\right)$ 15: **// Update deputy model*D*and Global model*G*** 16: $\:G=Aggregate\left(D\right)$ 17: $\:D={\theta\:}_{aggregated}$ 18: end for	

Additionally, a three-stage transfer learning strategy based on A-distance (ATTL), is designed to smooth knowledge from the global model to the local models, preventing performance regression during parameter updates. The pseudo-code of three-stage ATTL as shown in Algorithm 2. Here, $\:P$ is the personalized local model, $\:{l}_{supervised}$denotes the cross-entropy loss function.$\:{l}_{A-distance}$denotes the measurement algorithm for data distribution, and the A-distance algorithm was used in this study.

**Table:** 

Algorithm 2 Three-stage A-distance-based Transfer Learning (ATTL)	
1: Initialize Global model $\:G$, deputy model $\:D$, Personalized local model $\:P$ 2: for each communication round $\:t=0$ to $\:T-1$ do 3: **// Stage I**: $\:\varvec{P}$→$\:\varvec{D}$**transfer** 3: Fix $\:P$, update $\:D$: 4: $\:{l}_{D}={l}_{supervised}\left(D\right)+{l}_{A-distance}(P,D)$ 5: $\:\:\:D=Optimizer\left({l}_{D}\right)$ 6: // Stage transition condition 7: if AUC($\:D$) > AUC($\:P$) then 8: **// Stage II**: $\:\varvec{P}$**and**$\:\varvec{D}$**mutual learning** 9: Update $\:P$ and $\:D$: 10: $\:{l}_{D}={l}_{supervised}\left(D\right)+{l}_{A-distance}(P,D)$ 11: $\:D=Optimizer\left({l}_{D}\right)$ 12: $\:{l}_{P}={l}_{supervised}\left(P\right)+{l}_{A-distance}(D,P)$ 13: $\:\:D=Optimizer\left({l}_{D}\right)$ 14: end if 15: // Stage transition condition 16: if AUC($\:P$) > AUC($\:D$) then 17: **// Stage III**: $\:\varvec{D}$→$\:\varvec{P}$**transfer** 17: Fix $\:D$, update $\:P$: 18: $\:{l}_{P}={l}_{supervised}\left(P\right)+{l}_{A-distance}(D,P)$ 19: $\:\:P=Optimizer\left({l}_{P}\right)$ 20: end if 21: end for	

②**Personalized model loss for handling class imbalance in samples**.

The severe imbalance in data categories significantly affects the performance of model training. For the issue of class imbalance in federated learning, a personalized loss function is introduced, incorporating margin correction and contrastive prediction encoding.

Typically, for a classification task with $\:C$ classes, the corresponding cross-entropy loss function is:

1 $$\:{l}\left({x}_{i},{y}_{i};{\theta\:}_{f},{\theta\:}_{c}\right)=-\text{l}\text{o}\text{g}\left(\frac{\text{e}\text{x}\text{p}\left({\eta\:}_{{y}_{i}}\right)}{{\sum\:}_{c=1}^{C}\text{e}\text{x}\text{p}\left({\eta\:}_{c}\right)}\right)$$

Where $\:{x}_{i}$ is the $\:i$-th training data and $\:{y}_{i}$ is the ground truth. $\:{\theta\:}_{f}\:$is the parameter corresponding to the feature representation learning function, $\:{\theta\:}_{c}$ is the parameter corresponding to classifier function. $\:{\eta\:}_{c}$ is the $\:c$-th class prediction score from the linear classification function as follows:

2 $$\:{\eta\:}_{c}={\varvec{W}}_{c}\mathcal{z}+{\varvec{b}}_{\varvec{c}}$$

Where $\:{\varvec{W}}_{c}$ and $\:{\varvec{b}}_{\varvec{c}}$ represent the weight and bias matrix corresponding to the $\:c$-th class, $\:\mathcal{z}$ denotes the feature that has been encoded by the feature representation learning function.

For a classification task, the classification margin of the $\:c$-th class data point $\:{\mathcal{z}}_{1}$ is calculated as [16]:

3 $$\:{d}_{c}=\frac{{\varvec{W}}_{\varvec{c}}{\mathcal{z}}_{1}+{\varvec{b}}_{\varvec{c}}}{\left|\left|{\varvec{W}}_{\varvec{c}}\right|\right|}$$

Here, $\:\left|\left|\varvec{*}\right|\right|$ represents the L2-norm. ${z_1}$ is an arbitrary data point in the feature space. Thus, the prediction score $\:{\eta\:}_{c}$ can be expressed as $\:\left|\left|{\varvec{W}}_{\varvec{c}}\right|\right|{d}_{c}$.

Studies have shown that the decision boundary and prediction scores are correlated with the cardinality of each class, where the majority class tends to have a larger decision boundary [17, 18]. Thus, we aim to calibrate the margins to obtain balanced predictions. The adjusted margin distance is expressed as follows:

4 $$\>{\hat d_c}{\rm{ = }}\>\omega {\>_c} \cdot \>{d_c} + \beta {\>_c}$$

Here, $\:{\omega\:}_{c}$ and $\:{\beta\:}_{c}$ are margin calibration coefficients derived from the training samples, and $\:{d}_{c}$ is the margin after the standard training. Thus, the calibrated logit is computed as:

5 $$\:\begin{array}{l}\left|\left|{\varvec{W}}_{\varvec{c}}\right|\right|{\widehat{d}}_{c}=\left|\left|{\varvec{W}}_{\varvec{c}}\right|\right|({{\omega\:}_{c}\cdot\:d}_{c}+{\beta\:}_{c})\\\:\:\:\:\:\:={\omega\:}_{c}\cdot\:\left|\left|{\varvec{W}}_{\varvec{c}}\right|\right|{d}_{c}+{\beta\:}_{c}\left|\left|{\varvec{W}}_{\varvec{c}}\right|\right|\\\:\:\:\:\:\:={\omega\:}_{c}\cdot\:{\eta\:}_{c}+{\beta\:}_{c}\left|\left|{\varvec{W}}_{\varvec{c}}\right|\right|\end{array}$$

By combining Eqs. (1) and (5), the margin-based cross-entropy loss function is formulated as follows:

6 $$\:{{l}}_{\text{M}\text{A}\text{R}\text{C}}\left({x}_{i},{y}_{i};{\theta\:}_{r},{\theta\:}_{o}\right)=-\text{l}\text{o}\text{g}\left(\frac{\text{e}\text{x}\text{p}\left({\omega\:}_{{y}_{i}}{\eta\:}_{{y}_{i}+{\beta\:}_{{y}_{i}}\left|\right|{\varvec{W}}_{{y}_{i}}\left|\right|}\right)}{{\sum\:}_{c=1}^{C}\text{e}\text{x}\text{p}\left({\omega\:}_{c}{\eta\:}_{c+{\beta\:}_{c}\left|\right|{\varvec{W}}_{c}\left|\right|}\right)}\right)$$

It is worth noting that this study does not explicitly introduce the imbalance ratio into the margin correction function, but rather balances the margins of the two types of samples by constructing a margin correction function (Eq. 4). by minimizing the loss function (Eq. 6) and automatically obtaining the weight parameters $\:\omega\:$ and $\:\beta\:$ based on the training samples, the correction of the original margins is achieved.

In addition, to further mitigate the sample imbalance, this study extracts the fully connected layer parameters $\:{f}^{G}$ of the global model to represent global information. The fully connected layer parameters $\:{f}_{k}^{L}$ of the $\:k$-th local model are used to represent the information of the $\:k$-th local model. The contrastive predictive coding (CPC) technique is adopted to align the distance between the global model representation and the local model representations, constructing a regularization loss term to constrain the model’s learning process. The regularization loss is formulated as follows:

7 $$\:{\mathcal{l}}_{\text{C}\text{P}\text{C}}\left({f}^{G},{f}^{L}\right)=-\mathbb{E}\left[log\frac{s({f}^{G},{f}_{k}^{L})}{\sum\:_{i=1}^{K}s({f}^{G},{f}_{i}^{L})}\right]$$

Where $\:K$ is the number of clients, the symbol $\:\mathbb{E}$ denotes the expectation operator, and $\:\text{s}$ represents some kind of similarity measure function, and cosine similarity was used in this study.

Finally, the total loss is calculated as follows:

7 $$\>\ell oss = \>{\ell _{{\rm{MARC}}}} + \alpha \>*{\ell _{{\rm{CPC}}}}$$

Where $\:\alpha\:$ is the regularization parameter. This loss function guides local models to align with the globally balanced objective, thereby improving training performance. A detailed description is provided in Supplementary S2.

#### (2) construction of classifier

First, the convolutional kernels of the trained local model are utilized as feature extractors to generate feature maps for each CT image of every patient. Next, the average of all feature maps for each patient is computed to represent the patient’s final features, yielding a total of 7,616 federated learning features, as detailed in Supplementary S3. Subsequently, the maximum relevance minimum redundancy (mRMR) algorithm is employed to select the most significant features, effectively minimizing redundancy while maximizing discriminative power. Finally, a Bayesian extreme learning machine is applied to construct the final classification model.

### Evaluation and comparison of models

To comprehensively evaluate the performance of RFed in a multicenter environment, various comparative experiments were conducted. First, to validate the superiority of the proposed model, several state-of-the-art federated learning methods were used for comparison, including FedAvg [10], FedBn [19], FedProx [20], MetaFed [21], and PrrFed [15]. Additionally, following previous research and referencing related guidelines [22–24], this study constructed clinical models using the Bayesian extreme learning machine algorithm, incorporating features such as the longest diameter, smoking history, lobulated sign, spiculated sign, and CEA index.

To assess the generalizability of the proposed framework, classical network architectures—ResNet34 [25], VGG16 [26], Inception-v3 [27], and Vision Transformer (ViT) [28]—were used as base models for federated learning. Furthermore, out-of-distribution generalization experiments were conducted, where models trained on data from three centers were tested on a fourth center that did not participate in the training.

### Statistical analysis

To comprehensively evaluate the performance of various algorithms, this study utilized quantitative metrics such as the Area Under the Receiver Operating Characteristic Curve (AUC) and the F1 score to validate the predictive results. The F1 score, which is the harmonic mean of specificity and sensitivity, is primarily used to assess model performance in the context of imbalanced data. The Receiver Operating Characteristic (ROC) curve was employed to illustrate the overall performance of different methods. Furthermore, Decision Curve Analysis (DCA) was utilized to evaluate the clinical effectiveness of the model in predicting postoperative progression in early-stage NSCLC. These quantitative metrics and analyses offer a thorough assessment of the model’s performance and its clinical utility in predicting progression in early-stage NSCLC patients.

Additionally, to verify the degree of model improvement, Delong and Integrated Discrimination Improvement (IDI) tests were employed. Statistical analyses were performed using two-tailed tests, with a p-value of less than 0.05 considered statistically significant.

### Details of the algorithm parameters and software system

The details of the algorithm parameters are as follows: the base model used is ResNet34 with a batch size of 32. Communication occurs every 50 iterations, and local model perform 5 optimization iterations between communications. The learning rate is set to 0.01, and the loss function regularization parameter $\:\alpha\:$ is set to 0.6. Additionally, the shared low-frequency threshold $\:{r}_{max}$is set to 0.3. Specific ablation experiments for regularization parameter $\:\alpha\:\:$and low-frequency threshold $\:{r}_{max}\:$are shown in Supplementary S4.

The system is configured with Windows 11, featuring an NVIDIA RTX 4090 graphics card with 24GB of GPU memory, and utilizes CUDA 11.7 for accelerated computation. The system is powered by a 13th Gen Intel Core i9-13900 K CPU, delivering substantial processing power. For deep learning tasks, PyTorch 2.0.0 serves as the framework, with Python 3.10 as the programming environment.

## Results

### RFed exhibits superior diagnostic performance in early-stage NSCLC patients

#### Comparison with traditional clinical models

We evaluated the proposed RFed across the four centers and summarized the results in Table 2. The RFed outperforms traditional clinical models on multiple metrics. The average AUC of RFed is 0.923, which is 22.7% higher than that of the clinical model (average AUC = 0.696). The Delong test revealed significant differences between the clinical model and RFed with respect to AUC across the four centers (*p* = 0.052, 0.012, 0.004, 0.001). The IDI indicated that RFed exhibited significantly better prediction performance than the clinical model across the four centers (IDI = 0.314, *p* < 0.001; IDI = 0.187, *p* < 0.001; IDI = 0.148, *p* < 0.001; IDI = 0.427, *p* < 0.001).

**Fig. 2:**
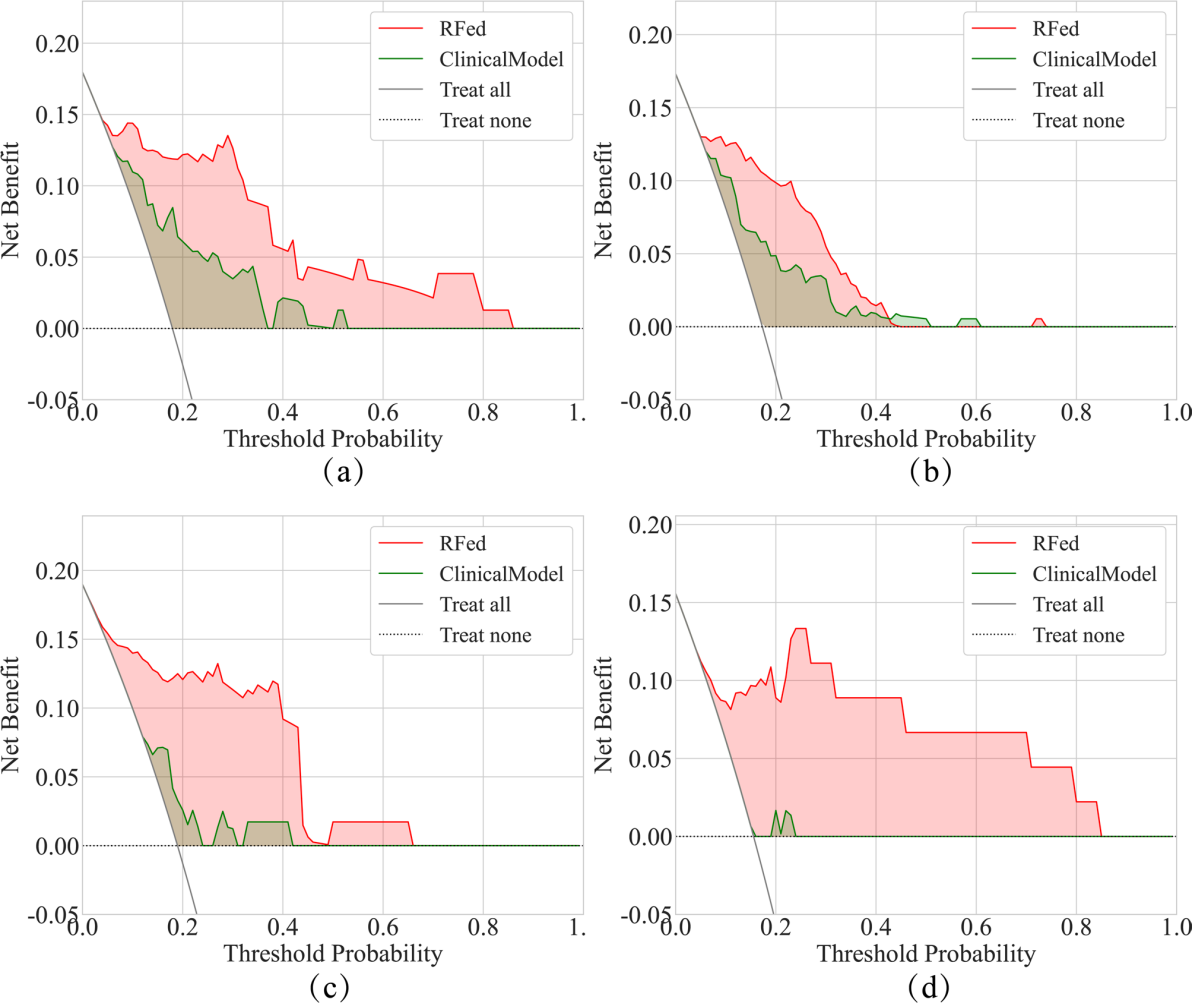
DCA curves in four centers. (**a**) Center (A) (**b**) Center (B) (**c**) Center (C) (**d**) Center (D) Notes: RFed, robust federated learning

To assess the clinical effectiveness of the model in predicting postoperative progression of early-stage NSCLC patients, DCA curves were plotted. The threshold ranges for the four centers are 0.04–0.99, 0.05–0.99, 0.02–0.99, and 0.05–0.99, respectively. This wide range of thresholds suggests that RFed has a higher net benefit in distinguishing between the LCP and LCNP groups compared to the clinical model.(Fig. 2).

**Fig. 3:**
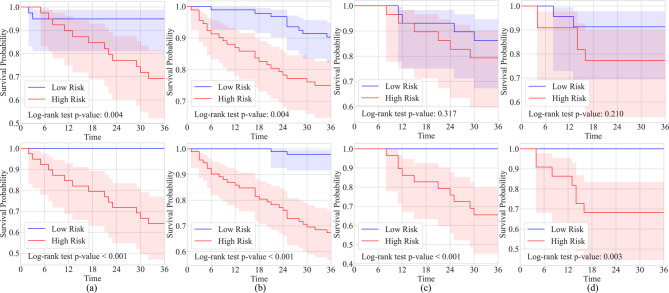
Kaplan-Meier curves of predicted high-risk (red) and low-risk (blue) groups. (**a**) Center (A) (**b**) Center (B) (**c**) Center (C) (**d**) Center (D) A p-value < 0.05 indicates statistical significance, and the shaded regions represent the confident intervals. The first row shows the Kaplan-Meier curves of clinical model, and the second row shows the Kaplan-Meier curves of the proposed federated learning framework. Time is measured in months

We further evaluated our method through statistical analysis, with the Kaplan-Meier curves presented in Fig. 3. The figure displays survival curve analyses for four centers, comparing the clinical model (first row) and the RFed model (second row). The Kaplan-Meier curves show the survival probabilities for the low-risk (blue line) and high-risk (red line) groups. The Log-rank test p-values indicate significant survival differences between the groups, with the RFed model consistently demonstrating clearer separation between the low-risk and high-risk groups compared to the clinical models. In addition, this study integrated age, gender, longest diameter, CEA, and RFed factors performed univariate and multivariate Cox regression analyses, and calculated hazard ratios with 95% confidence intervals, and as shown in Supplementary S5. In univariate Cox regression analysis, the HR (95% CI) for RFed was 4.034 (2.189–7.435) with a p-value of less than 0.001. In multivariate Cox regression analysis, the HR (95% CI) for RFed was 9.176 (2.835–29.693) with a p-value of less than 0.001. The results showed that RFed increased the independent prognostic value after adjusting for age, gender, longest diameter and CEA, and RFed outperformed clinical model in predicting postoperative risk of early-stage NSCLC patients.

**Fig. 4:**
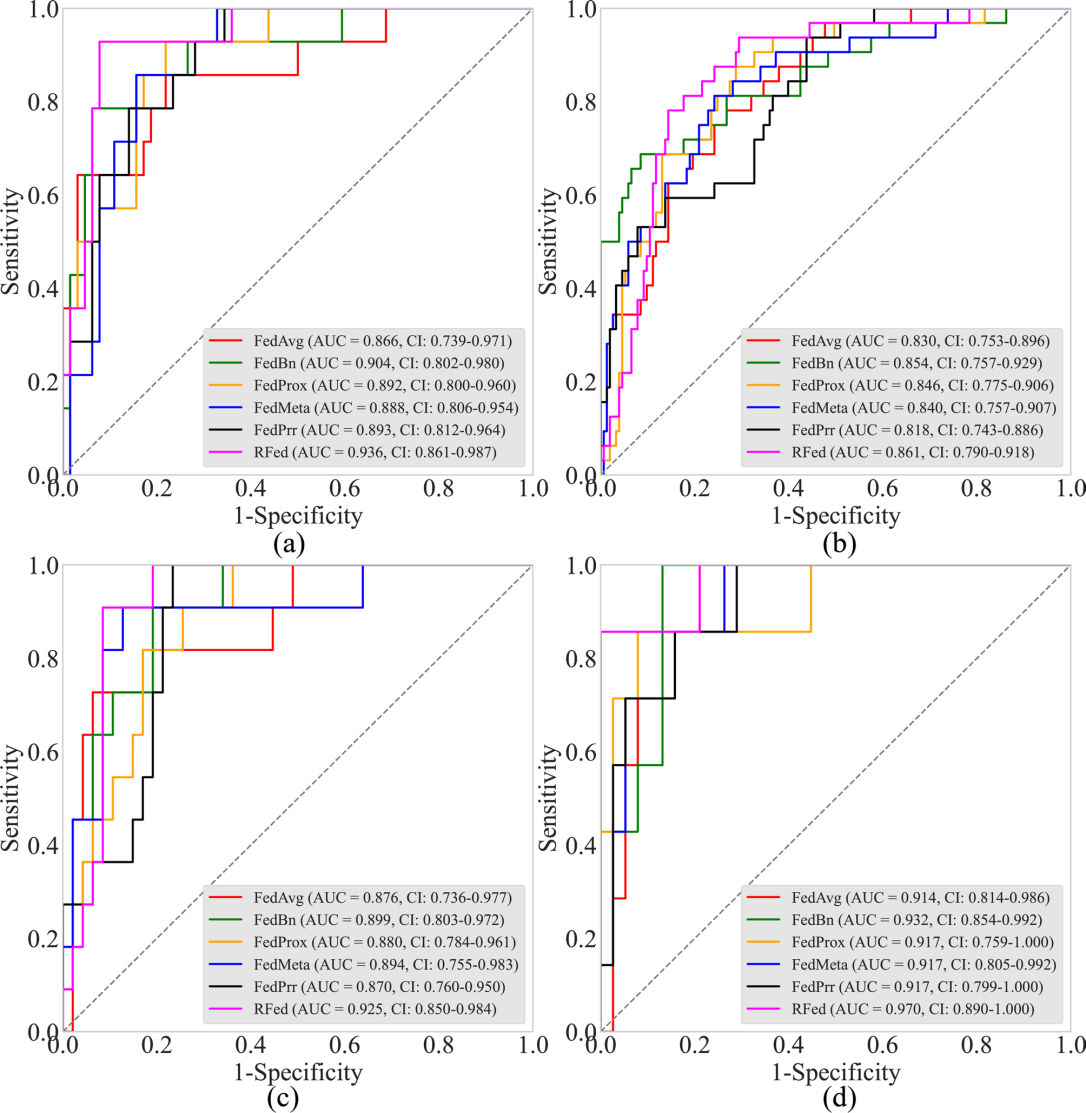
The receiver operating characteristic curve of various federated learning algorithms. (**a**) Center (A) (**b**) Center (B) (**c**) Center (C) (**d**) Center (D) Notes: RFed, robust federated learning. AUC, area under the receiver operating characteristic curve. CI, confidence interval

**Comparison with state-of-the-art federated learning models**: The results of our study demonstrate that the proposed RFed exhibits higher diagnostic efficiency when applied to data from the four centers, as shown in Table 3; Fig. 4. The AUCs obtained from the four centers are as follows: 0.936, 0.861, 0.925, and 0.970, respectively. When comparing the performance of RFed to state-of-the-art federated learning models, we observed an overall improvement in the average AUC by 2.90–5.84%. Additionally, the F1 score of RFed is relatively high overall (average F1 score: 0.598), reflecting its advantage in handling imbalanced datasets, while its performance is slightly weaker than that of MetaFed.

**Table 2:** Evaluation of performance between RFed and various models

Method	AUC					F1 score				
	A	B	C	D	Mean ± SD	A	B	C	D	Mean ± SD
Clinical Model	0.790	0.747	0.651	0.594	0.696 ± 0.089	0.476	0.371	0.276	0.353	0.369 ± 0.082
FedAve	0.866	0.830	0.876	0.914	0.872 ± 0.030	0.533	0.489	0.640	0.615	0.569 ± 0.061
FedBn	0.904	0.854	0.899	0.932	0.897 ± 0.028	0.600	0.638	0.375	0.609	0.556 ± 0.105
FedProx	0.892	0.846	0.880	0.917	0.884 ± 0.026	0.520	0.525	0.593	0.546	0.546 ± 0.029
MetaFed	0.888	0.840	0.894	0.917	0.884 ± 0.028	0.588	0.514	0.750	0.632	0.621 ± 0.086
PrrFed	0.893	0.818	0.870	0.917	0.875 ± 0.037	0.629	0.434	0.552	0.667	0.571 ± 0.089
RFed	0.936	0.861	0.925	0.970	0.923 ± 0.039	0.553	0.595	0.611	0.632	0.598 ± 0.029

Note: RFed, robust federated learning. AUC, area under the receiver operating characteristic curve. SD: standard deviation

### RFed has good robustness and generalizability

**Evaluation of reliability and targeted diagnostic utility**: In this study, the sensitivity of the model was analyzed by fixing the specificity case, as shown in Table 3. Centers A and D maintained stable sensitivity until higher specificity demands, while B and C showed greater variance. All achieved > 85% sensitivity at 80% specificity (D: 85.7%). However, at the 90% specificity threshold, Hospital A had the highest sensitivity (78.6%), B and C showed moderate results (43.8% and 36.4%), and D achieved 0% sensitivity. This limitation at stringent specificity likely stems from opportunities for model refinement and the limited dataset size. Future research will expand the case cohort and develop more robust diagnostic models to address these issues. In addition, this study pooled the predicted values from the four centers and assessed the calibration performance of the model by plotting the calibration curves, as shown in Supplementary S6. The results showed that the predicted probabilities were basically consistent with the actual observed probabilities, and the overall calibration slope was 0.892, which was close to the ideal value of 1, indicating that the predicted probabilities were reasonably calibrated, trustworthy, and could be used for subsequent clinical interpretation.

**Table 3:** Sensitivity analysis under fixed specificity

Center	Specificity			
	0.6	0.7	0.8	0.9
A	0.929	0.929	0.786	0.786
B	0.938	0.906	0.812	0.438
C	0.909	0.909	0.909	0.364
D	0.857	0.857	0.857	0.000

#### Out-of-distribution generalization of the proposed framework

To further validate the generalization ability of RFed, we also used datasets A, B, C, and D as separate testing sets, with the remaining datasets serving as training sets in each case, as shown in Table 4. The results demonstrate that the proposed federated learning framework achieves consistent and robust performance across different train-test configurations. The AUC values range from 0.770 ± 0.128 to 0.818 ± 0.009, reflecting strong discriminatory ability across all configurations. Similarly, the F1 score, ranging from 0.429 ± 0.038 to 0.473 ± 0.031, indicates reliable performance in balancing specificity and sensitivity across varying data distributions. These findings highlight the model’s strong generalization ability and adaptability to unseen datasets, validating the effectiveness of the federated learning framework in utilizing multi-center data for early-stage NSCLC prognostic diagnosis.

**Table 4:** Performance of the federated learning framework on different train-test configurations

Case	Training set	Testing set	AUC	Mean ± SD	F1 score	Mean ± SD
Case 1	A	D	0.829	0.818 ± 0.009	0.366	0.373 ± 0.048
	B	D	0.819		0.318	
	C	D	0.806		0.435	
Case 2	A	C	0.768	0.770 ± 0.128	0.381	0.429 ± 0.038
	B	C	0.787		0.475	
	D	C	0.756		0.430	
Case 3	A	B	0.780	0.776 ± 0.007	0.473	0.460 ± 0.009
	C	B	0.766		0.456	
	D	B	0.782		0.451	
Case 4	B	A	0.800	0.783 ± 0.012	0.431	0.473 ± 0.031
	C	A	0.777		0.504	
	D	A	0.773		0.485	

Note: AUC, area under the receiver operating characteristic curve. SD: standard deviation

#### Effectiveness of the federated learning framework across different architectures

To validate the effectiveness of the proposed federated learning framework, three convolutional neural networks (ResNet34, VGG16, and Inception V3) and a transformer-based backbones (ViT) were chosen as base models to construct the framework, as shown in Table 5. The AUC values across all models demonstrate strong discriminatory ability, with average AUC ranging from 0.882 ± 0.054 (ViT) to 0.919 ± 0.039 (ResNet34), indicating consistently high performance across different datasets. In terms of F1 score, Inception V3 achieved the highest mean F1 score (0.630 ± 0.073), reflecting its superior ability to balance precision and recall. ResNet34 followed with an average F1 score of 0.613 ± 0.029, demonstrating stable performance. Overall, these results indicate that the proposed federated learning framework performs effectively regardless of the underlying base model. These findings validate the robust ability of the federated learning framework across different base architectures and datasets.

**Table 5:** Performance of different base models in the federated learning framework across centers

Method	Evaluation	Center A	Center B	Center C	Center D	Mean ± SD
VGG16	AUC	0.917	0.888	0.919	0.940	0.916 ± 0.019
	F1 score	0.571	0.456	0.688	0.250	0.465 ± 0.162
Inception V3	AUC	0.920	0.850	0.923	0.966	0.913 ± 0.042
	F1 score	0.714	0.536	0.640	0.714	0.630 ± 0.073
ResNet34	AUC	0.936	0.861	0.925	0.970	0.919 ± 0.039
	F1 score	0.553	0.595	0.611	0.632	0.613 ± 0.029
ViT	AUC	0.913	0.824	0.851	0.940	0.882 ± 0.054
	F1 score	0.692	0.492	0.421	0.250	0.464 ± 0.183

Note: AUC, area under the receiver operating characteristic curve. SD: standard deviation

**Fig. 5:**
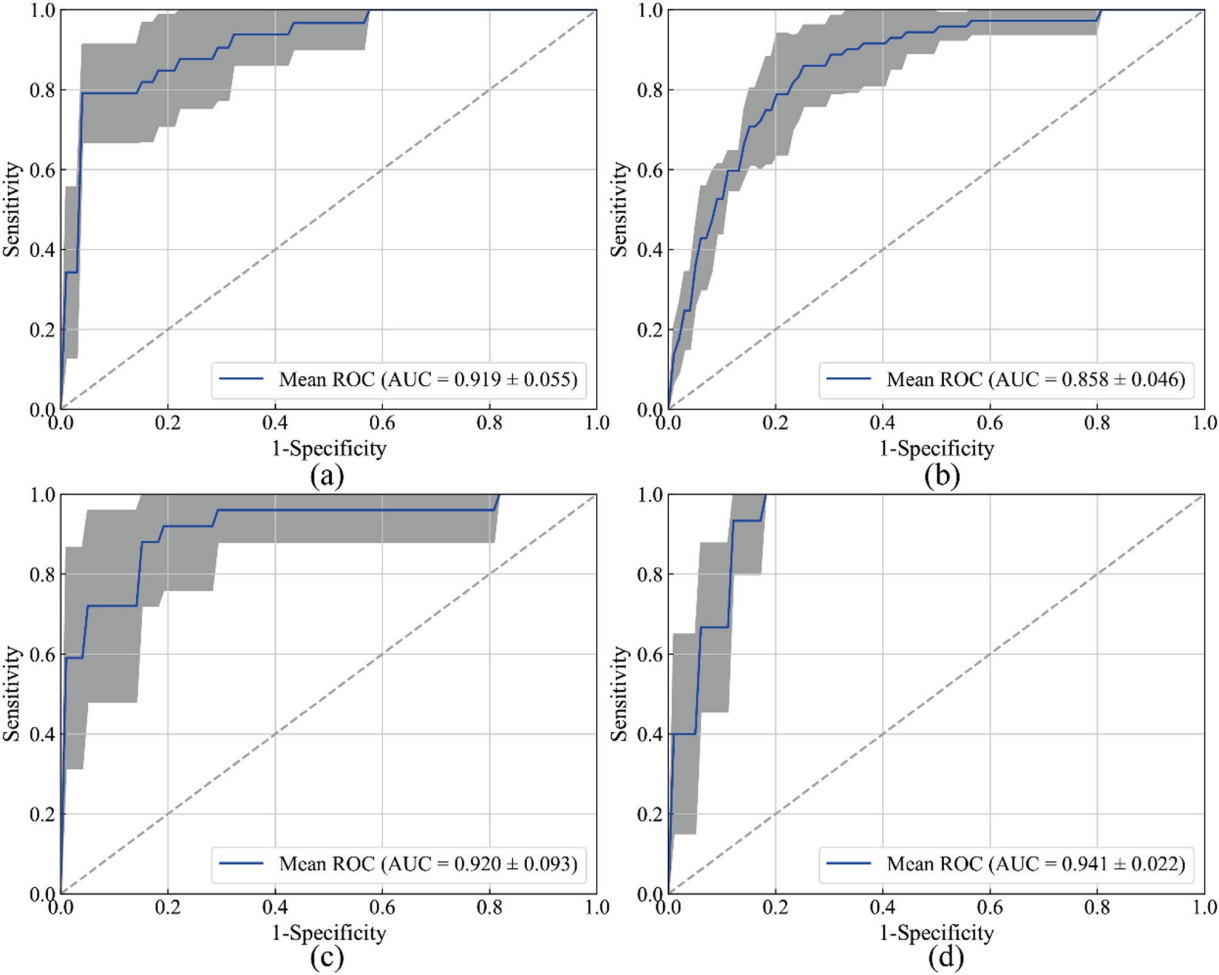
Four center five-fold cross-validation ROC curves. (**a**) Center (A) (**b**) Center (B) (**c**) Center (C) (**d**) Center (D) The blue curve is the AUC average of the five-fold curve ROC. The gray area is the upper and lower limits of the ROC curve. Notes: AUC: Area under the curve. ROC: Receiver operating characteristics

To further verify the robustness of the model, the results of multi-center RFed were assessed using five-fold cross-validation. The average AUC results for cross-validation across the testing sets of four centers were 0.919, 0.858, 0.920, and 0.941, respectively. The cross-validation AUC results are shown in Fig. 5.

#### Performance comparison of federated learning methods on other cancer data

To further validate the effectiveness of the proposed RFed algorithm, we applied it to skin cancer data alongside other federated learning methods, and the detailed information of data refers to reference [15]. The results, as shown in Table 6, demonstrate that RFed outperforms all other methods in both AUC and F1 score across all centers. Specifically, RFed achieved a mean AUC of 0.908 ± 0.035 and a mean F1 score of 0.757 ± 0.056, outperforming the next best method, MetaFed, in both metrics. These results highlight RFed’s superior performance in handling imbalanced datasets and its robustness across diverse data distributions.

**Table 6:** Analysis of performance on skin cancer data

Method	AUC					F1 score				
	A	B	C	D	Mean ± SD	A	B	C	D	Mean ± SD
FedAve	0.815	0.823	0.761	0.711	0.778 ± 0.045	0.574	0.481	0.568	0.442	0.516 ± 0.056
FedBn	0.831	0.964	0.800	0.814	0.852 ± 0.065	0.550	0.721	0.547	0.621	0.610 ± 0.071
FedProx	0.817	0.701	0.768	0.748	0.759 ± 0.042	0.507	0.391	0.547	0.460	0.476 ± 0.058
MetaFed	0.924	0.958	0.903	0.843	0.907 ± 0.042	0.782	0.803	0.747	0.677	0.752 ± 0.048
PrrFed	0.891	0.982	0.855	0.841	0.892 ± 0.055	0.719	0.853	0.684	0.656	0.728 ± 0.076
RFed	0.947	0.930	0.900	0.856	0.908 ± 0.035	0.833	0.789	0.709	0.697	0.757 ± 0.056

Note: AUC, area under the receiver operating characteristic curve. SD: standard deviation

### RFed has good feature extraction and interpretable capabilities

**Fig. 6:**
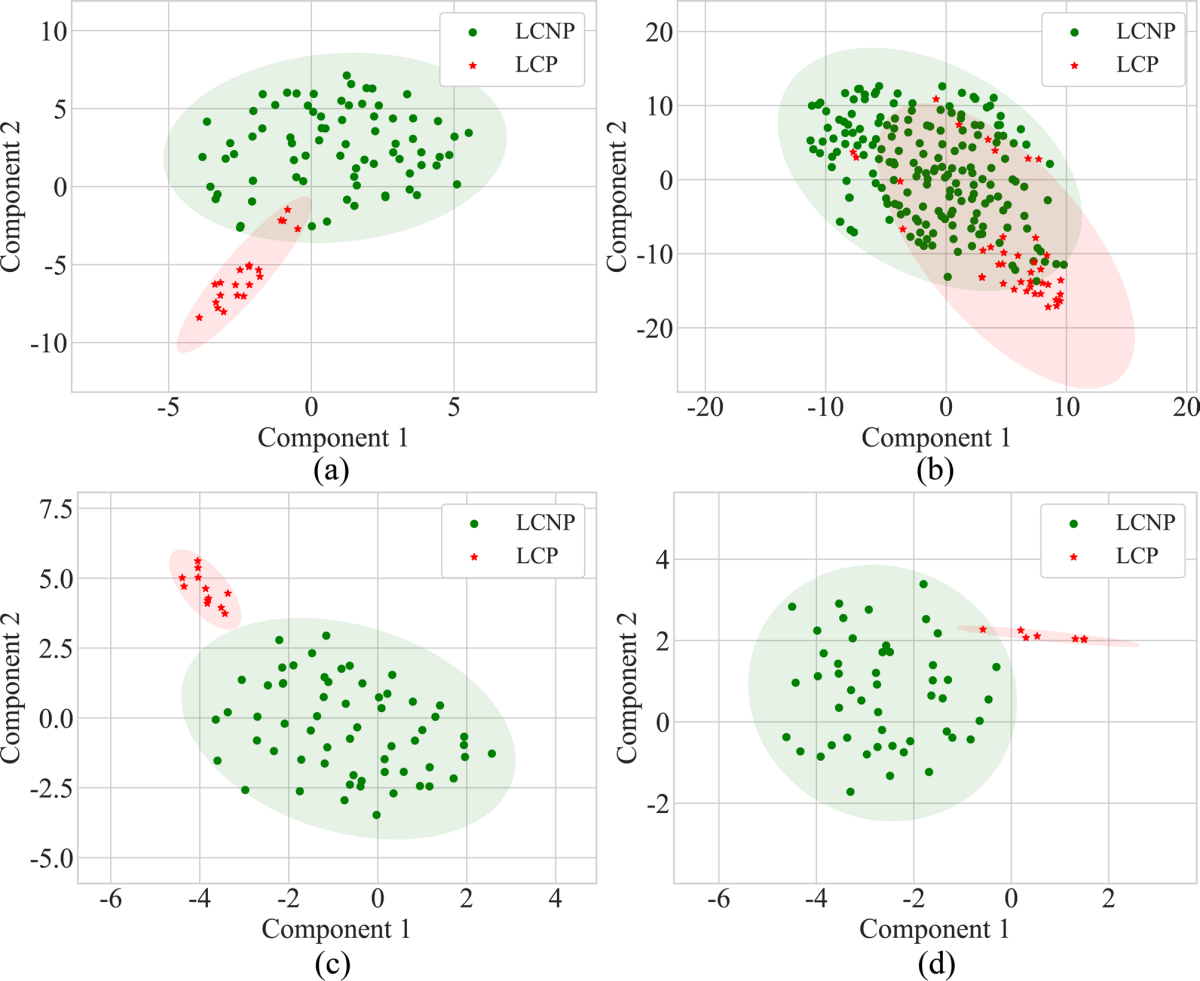
t-SNE visualization of class distributions across centers. (**a**) Center (A) (**b**) Center (B) (**c**) Center (C) (**d**) Center (D) Notes: LCP: lung cancer progression. LCNP: lung cancer non-progression

To assess and visualize the effectiveness of feature representation, we employed the t-SNE method [29]. As shown in Fig. 6, the first row of the visualization illustrates the distribution of raw data, while the second row shows the processed feature representations after applying RFed. In the first row, it is evident that there is variability in the separation between the two classes (LCNP and LCP) across the centers in the raw data. In certain centers, such as Center A and Center D, the two classes show partial overlap, while in others, like Center B and Center C, the overlap is more pronounced, suggesting that the raw data lacks clear separability in some cases. However, after applying the federated learning framework, as shown in the second row, the class separability improves significantly across all centers. This enhancement demonstrates the ability of the federated learning framework to improve feature representation and make it more robust to data variations from different centers.

Exploring multi-center features helps elucidate the interactions and dependencies among features. In the federated learning framework, due to the heterogeneity of multi-center data, the center-specific features learned by local models from their respective local data exhibit biases and center-specific traits tailored to local tasks. Simultaneously, as model parameters are exchanged between centers, center-common features that are adaptable to multi-center data also emerge. To illustrate these center-specific and center-common features and their relationships, feature correlation heatmaps were generated with reference to the approach of article [30].

**Fig. 7:**
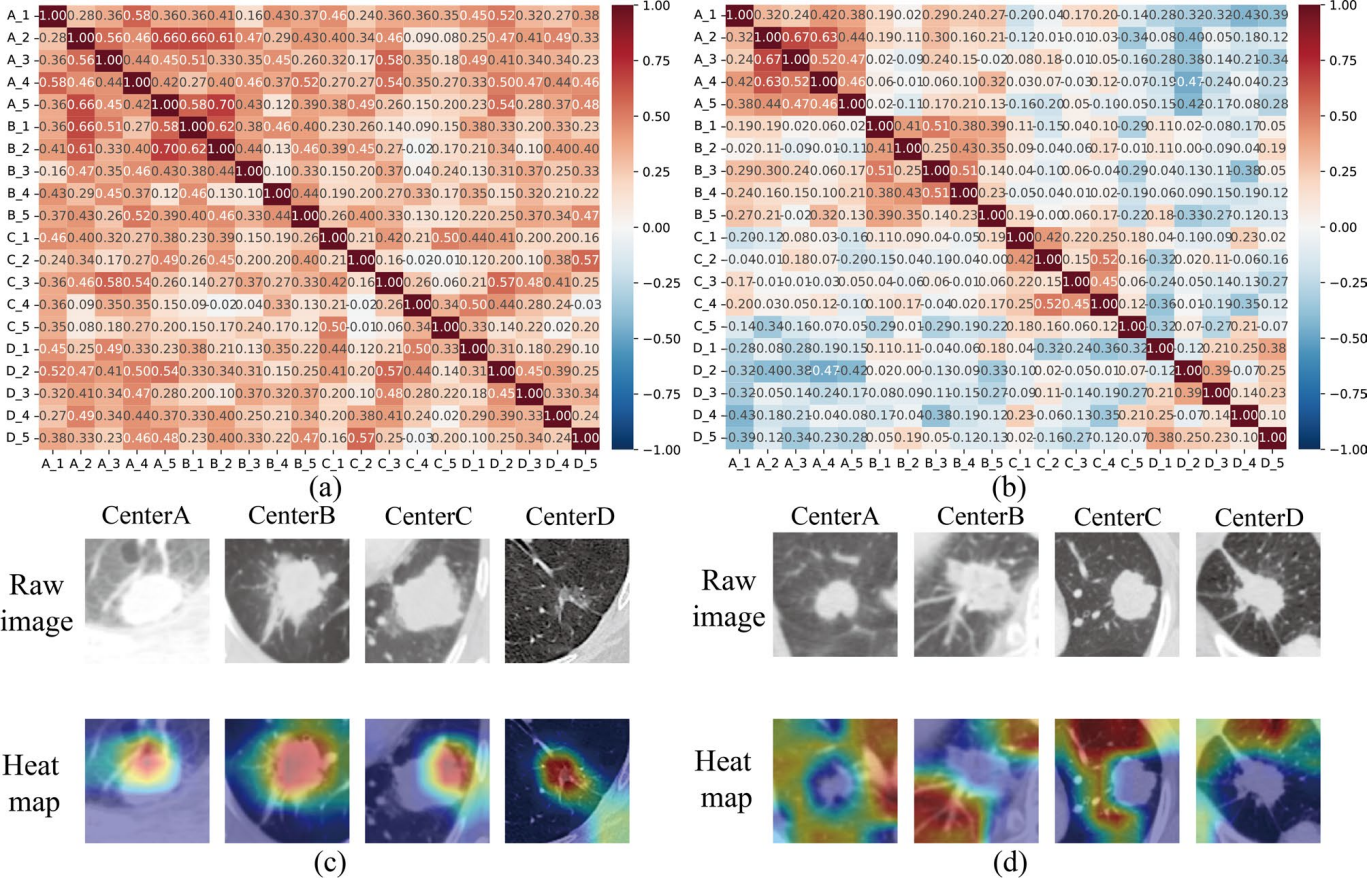
Heat map analysis. (**a**) Center-common feature of lung cancer progression group. (**b**) Center-specific features of lung cancer progression group. (**c**) Class activation mapping of lung cancer progression group. (**d**) Class activation mapping of lung cancer non-progression group. Notes: A_1, the first feature from center A. Red areas indicate a high level of model attention, while blue areas indicate a low level of model attention

The results are shown in Fig. 7a for center-common features of lung cancer progression group and Fig. 7b for center-specific features of lung cancer progression group. In Fig. 7a, high correlations are observed across different centers, while the correlations within the same center are relatively lower. This pattern indicates that the center-common features capture global characteristics consistent across centers, making them well-suited for cross-center feature sharing and collaborative learning. In contrast, Fig. 7b illustrates the center-specific features, where high correlations are evident within the same center, while correlations between different centers are notably lower. For the correlation heat map related to lung cancer non-progression group, please refer to Supplementary S7. This suggests that the center-specific features reflect local characteristics unique to each center, enabling the model to effectively capture and preserve center-specific information. In addition, to investigate the interpretability and classification basis of the federated learning features for the two group, we conducted category visualization by class activation mapping [31]. In Fig. 7c and d, we present the federated feature visualization images of eight cases, including both progression group and non-progression group. The visualizations highlight that the federated learning features exhibit a higher lesion focus for the progression group compared to the non-progression group.

## Discussion

Prognosis assessment of early-stage NSCLC is critical for improving patient outcomes by enabling timely intervention. However, in clinical practice, challenges such as data heterogeneity across multiple centers and class imbalance between positive and negative samples complicate the predictive modeling for early-stage NSCLC. To address these challenges, this study proposes a robust federated learning framework (RFed). The model exhibits exceptional diagnostic performance, strong generalizability, and effective feature extraction and interpretability capabilities. This approach has significant implications for assisting clinicians in assessing the risk of postoperative progression in early-stage NSCLC, thereby facilitating the development of personalized treatment plans tailored to individual patients.

The first key finding of this study is that the proposed RFed model can effectively associate preoperative CT images with the postoperative progression of early-stage NSCLC patients, enabling accurate assessment of the risk of recurrence or metastasis. RFed excels in handling multicenter heterogeneous data and integrating high-dimensional imaging features, allowing it to assess risks of postoperative progression that are challenging to detect visually. RFed outperformed all comparators by an average AUC of 0.923 ± 0.039 across four centers (Table 2). This performance is further validated by significant improvements in the DCA and Kaplan-Meier curves (Figs. 2 and 3), which show higher net benefits across a range of threshold probabilities (0.02 to 0.99). These results underscore the potential of RFed to enhance clinical decision-making, particularly in cases where traditional models may be less effective. Additionally, RFed’s ability to integrate diverse, heterogeneous datasets from multiple centers highlights its potential for multi-center collaborations, offering a promising solution to challenges like data privacy, which are common in real-world clinical settings.

The second key finding is that the RFed model demonstrates strong robustness and generalization, ensuring reliable postoperative risk prediction across diverse clinical settings. In the out-of-distribution generalization test, RFed maintained consistent AUC values (ranging from 0.770 ± 0.128 to 0.818 ± 0.009) and F1 scores (from 0.429 ± 0.038 to 0.473 ± 0.031) across independent test sets (Table 4). This highlights RFed’s ability to adapt to unseen data distributions, a critical feature for clinical deployment. Additionally, RFed showed flexibility when evaluated across different convolutional neural network architectures (ResNet34, VGG16, and Inception V3), with ResNet34 achieving the highest mean AUC of 0.919 ± 0.039 (Table 5). These findings emphasize RFed’s robustness and generalization, making it adaptable to various computational environments and suitable for different healthcare applications. This strong capability ultimately boosts clinical confidence in the model and enhances its potential for widespread use in personalized treatment planning and early disease screening.

The third key finding of this study underscores the RFed model’s feature representation and enhanced interpretability. T-SNE analysis demonstrated that the proposed federated learning framework significantly improved class separability across multiple centers. While the raw data exhibited variability and overlap between the two classes (LCNP and LCP), particularly in certain centers, the RFed model refined feature representations, resulting in clearer class distinctions (Fig. 6). Further analysis of multi-center features revealed that the model effectively learned both center-common features and center-specific features (Fig. 7a and b). The ability to simultaneously capture global (center-common) and local (center-specific) information enhances the model’s interpretability, providing transparent insights into the underlying factors driving predictions. In addition, by class activation mapping, the visualizations highlight that the federated learning features exhibit a higher lesion focus for the progression group compared to the non-progression group (Fig. 7c and d). This clarity enables clinicians to better understand the rationale behind risk assessments, thereby fostering trust in the model’s decisions. Consequently, this interpretability not only supports more informed and confident clinical decision-making but also ensures the model’s adaptability and relevance across diverse clinical settings, all while maintaining personalized and explainable predictions tailored to each center’s unique data characteristics.

Despite its promising results, this study has several limitations. The sample size, constrained by the availability of multi-center datasets, may limit the generalizability of the findings to broader populations. In addition, the current study only used data from a single modality, and the next step is to consider the introduction of multimodality to construct more effective diagnostic models. In addition, there are still some limitations in the current risk threshold delineation, and in the next study, we will investigate a more refined delineation of the thresholds, and even consider introducing more clinical information to further modify our thresholds and improve their clinical applicability. Addressing these limitations by expanding datasets, exploring new clinical applications, and optimizing computational efficiency will be key directions for future research.

## Conclusion

The robust federated learning model effectively predicts the risk of postoperative progression in early-stage non-small cell lung cancer, offering significant clinical value for enhancing stratified management and developing precise treatment strategies for early-stage lung cancer patients.

## Electronic supplementary material

Below is the link to the electronic supplementary material.

Supplementary Material 1

## Data Availability

The original contributions presented in the study are included in the article/Supplementary Material. Further inquiries can be directed to the corresponding authors.

## Abbreviations

NSCLC: Non-small cell lung cancer
RFed: Robust federated learning model
AUC: Area under the curve
DCA: Decision curve analysis
DL: Deep learning
FL: Federated learning
CT: Computed tomography
PACS: Picture archiving and communication system
LCP: Lung cancer progression
LCNP: Lung cancer non-progression
CEA: Carcinoma embryonic antigen
ROI: Region of interest
EPFA: Exponential progressive fourier aggregation
ATTL: A-distance-based transfer learning
mRMR: Maximum relevance minimum redundancy
ROC: Receiver operating characteristic
IDI: Integrated discrimination improvement
