# Supplementary material for: Collaborative assessment of the risk of postoperative progression in early-stage non-small cell lung cancer: a robust federated learning model
Source: Cancer Imaging. 2025 Jul 18;25:92. doi: 10.1186/s40644-025-00911-y (PMC12273366; doi:10.1186/s40644-025-00911-y)
Supplement: Supplementary file 1 — Supplementary Material 1 [file 40644_2025_911_MOESM1_ESM.docx]

**Supplemental Materials**

**Supplementary S1: CT scanning parameters and data preprocessing process**

**CT scanning parameters:** The four research centers use one of the following four CT scanners: Siemens Definition Force, Siemens Somatom 16, Toshiba Aquilion 64, and GE Discovery 64. They employed helical CT volumetric scanning technology with the following scan parameters: Tube voltage: 120 kVp, Automatic tube current technique (40-150 mAs), Pitch: 0.75-1.0, Matrix size: 512 × 512, Field of view (FOV): 200 × 200 mm. The scanning range encompassed both lung apices to the level of the adrenal glands. Patients were positioned in a supine manner with both arms raised, and the scan was initiated at the end of a deep inspiration breath-hold. To obtain contrast-enhanced images, an injector with a flow rate of 2.5-3.5 mL/s was used to intravenously administer iodixanol (350-370 mgI/mL). CT scans were performed 25 seconds (arterial phase) and 50 seconds (venous phase) after injection. The cross-sectional enhanced venous phase CT images were selected for the study. Reconstructed images had a cross-sectional thickness of 0.625-1.50 mm with a 20%-30% overlap. For coronal and sagittal reconstructions, the slice thickness was 2.5-3.0 mm. It is worth noting that in this study, the window level for CT images in all four data centers is set at -400, and the window width is fixed at 500.

**Data preprocessing process**: The preprocessing steps for CT images in the data center include the following procedures: In order to accommodate the inputs of the deep learning model and to fully utilize its automatic feature extraction capabilities, this study used a bounding box for coarse segmentation of the ROIs and did not use pixel-level labeling. Firstly, a radiologist with over 10 years of experience in chest radiology manually delineates the contours of the tumors. Next, a rectangular box is created to encompass all tumor contours, serving as the Region of Interest (ROI), and then fully reviewed and validated by a second independent radiologist. Subsequently, all ROI images are resized to a rectangular shape of 128×128 pixels to meet the input requirements of the deep learning model. Therefore, the selection of the ROI box is less influenced by the clinical doctor's experience. The specific processing flow is shown in Figure S1.


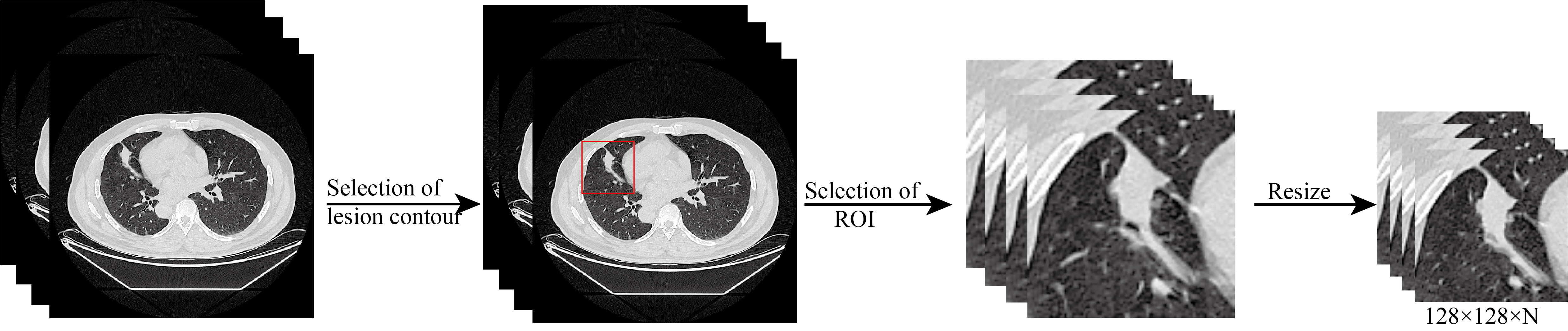


Figure S1 The processing flow of image

**Supplementary S2: Construction of a federated learning-based feature extraction model**

This algorithm can be divided into two parts: (1) Model parameter aggregation and download strategy. (2) Personalized model loss under samples class imbalanced

**(1) Model parameter aggregation and download strategy**

Traditional parameter aggregation and download strategy typically involve direct or weighted averaging of model parameters from multiple centers before sending them back. However, since the parameters from different client models may represent distinct semantic patterns at the same position, heterogeneity arises. Direct aggregation and backpropagation make it challenging to achieve model personalization. As a result, this study separately investigates strategies for model parameter aggregation and downloading.

**Fourier Transform-based Frequency Domain Parameter Aggregation**

Research has shown that the low-frequency components of data capture the underlying common information, while the high-frequency components represent more specific information unique to different datasets. Therefore, this study adopts a partially personalized Exponential Progressive Fourier Aggregation (EPFA) strategy, where Fourier Transform is used to share only the low-frequency parameters of the model, while the high-frequency parameters remain localized, thereby achieving a partially personalized federated strategy.

Considering that the convolutional layer of each model is a 4-dimensional parameter tensor, for the convenience of subsequent parameter analysis and transformation, this study first converts the 4-dimensional tensor into a 2-dimensional matrix $\omega_{k}\in{\mathbb{\mathbb{R}}}^{h_{1}d_{1}\times h_{2}d_{2}}$​, where $d_{1}$and $d_{2}$​ represent the number of input and output channels, and $h_{1}$and $h_{2}$are the corresponding sizes of the convolutional kernels. Based on the Fast Fourier Transform (FFT) algorithm, the frequency domain transformation is achieved as follows:

$\mathcal{\mathcal{F}}\left( \omega_{k} \right)\left( m,n \right)=\sum_{x,y} \omega_{k}\left( x,y \right)e^{-\mathbf{j}2\pi(\frac{x}{h_{1}d_{1}}m+\frac{y}{h_{2}d_{2}}n)}$ （1）

where $\mathbf{j}^{2}=-1$, and $m$ and $n$ represent the size of the 2-dimensional matrix after the tensor transformation, while $x$ and $y$ are the positions of specific elements.

To extract the low-frequency components of the aggregation information, this study uses a low-frequency mask matrix $M$ that changes with iterations, as expressed below:

$M\left( m,n \right)=\boldsymbol{1}_{\left( m,n \right)\in[-rh_{1}d_{1}:h_{1}d_{1},-rh_{2}d_{2}:h_{2}d_{2}]}$ （2）

where $r\in(0,0.5)$ is the threshold for the low-frequency components. Based on the above threshold, an averaging aggregation strategy is applied to aggregate the amplitude ${\mathcal{\mathcal{F}}}_{A}$ and phase ${\mathcal{\mathcal{F}}}_{P}$ of the parameters of the $k$-th client model, with the following formulas:

${\hat{\mathcal{\mathcal{F}}}}_{A}=\left( 1-M \right)* {\mathcal{\mathcal{F}}}_{A}\left( \omega_{k} \right)+\frac{1}{K}\sum_{i=1}^{K} M*{\mathcal{\mathcal{F}}}_{A}\left( \omega_{i} \right)$ （3）

${\hat{\mathcal{\mathcal{F}}}}_{P}=\left( 1-M \right)* {\mathcal{\mathcal{F}}}_{P}\left( \omega_{k} \right)+\frac{1}{K}\sum_{i=1}^{K} M*{\mathcal{\mathcal{F}}}_{P}\left( \omega_{i} \right)$ （4）

where $*$ denotes element-wise multiplication. Inspired by the common knowledge learned during the early stages of network training and the abstract deep information learned in later stages, this study adopts an iterative threshold learning method that changes in the form of an exponent, i.e., $r=\frac{r_{max}}{iter-t+1}$​, where $r_{max}$ represents the maximum value of the low-frequency component threshold, $iter$ is the maximum number of iterations, and $t$is the current iteration number.

Finally, the Fourier inverse transform is applied to convert the amplitude and phase components back into time-domain parameters, making them ready for subsequent parameter backpropagation. Additionally, considering the nature of fully connected layer parameters, no tensor transformation is required. Instead, a direct Fourier transform is performed, followed by low-frequency parameter aggregation and backpropagation.

**Parameter downloading Based on A-distance Transfer Learning Technique**

EPFA, from the frequency domain perspective, aggregates low-frequency information, which can overcome the performance degradation problem caused by time-domain parameter aggregation in the full model. However, through practical experiments, it was found that directly replacing the local model parameters with the aggregated global model parameters leads to a regression in the model's performance from the previous training, further interfering with the optimization in the next iteration. To overcome this issue, this study designs a three-stage transfer learning strategy based on A-distance[1], called A-distance-based Transfer Learning (ATTL), which transfers from the global model (G) through the deputy model (D) back to the personalized local model (P).

**Stage I**: When the knowledge obtained from the global model G is directly transmitted to the transition model T, the performance of the transition model degrades due to direct backpropagation of the information. Therefore, this study adopts a transfer learning strategy, where the personalized local model parameters are treated as source domain data and the transition model parameters as target domain data for adaptation learning, improving the diagnostic performance of the deputy model D. At this stage, the loss function is used to optimize the personalized local model P for independent local training, while the deputy model D is optimized using the following loss function:

${\mathcal{l}oss}_{D}\mathcal{=l}oss+{\mathcal{l}oss}_{A-distance}(p_{P}||p_{D})$ （5）

where $\mathcal{l}oss$ is the supervised loss under local data, using the personalized loss based on margin correction and contrastive prediction encoding proposed in this study. $p_{P}$​ and $p_{D}$​ represent the prediction probabilities of the personalized local model P and the deputy model D, respectively. ${\mathcal{l}oss}_{A-distance}(p_{P}||p_{D})$ is the distribution discrepancy loss term based on A-distance, which is mainly used to measure the probability distribution difference between $p_{P}$​ and $p_{D}$​, constraining and improving the predictive performance of the deputy model.

**Stage II**: As Stage I progresses, the performance of the transition model gradually approaches that of the personalized local model, i.e., AUC(T)>AUC(P), where AUC (Area Under the Curve) is the main metric for evaluating model performance. At this point, P and D become source domains for mutual learning, and model training is performed to exchange the global knowledge of the transition model with the personalized local knowledge of the local model. The deputy model D is optimized using formula (5), and the personalized local model is optimized using the following loss function:

${\mathcal{l}oss}_{P}\mathcal{=l}oss+{\mathcal{l}oss}_{A-distance}(p_{D}||p_{P})$ （6）

Through the mutual learning strategy, the global knowledge from the server can be smoothly transferred to the personalized local model P, enhancing the stability of the client model.

**Stage III**: As the optimization continues, when the diagnostic performance of the transition model TTT exceeds that of the personalized local model, the parameters of the transition model are treated as source domain data and the parameters of the personalized local model as target domain data for adaptation learning. The deputy model D is optimized using the loss function $\mathcal{l}oss$, and the personalized local model P is optimized using formula (6). Through this process, the knowledge from the global model can be maximally transferred to the local model, achieving effective parameter backpropagation and avoiding performance regression caused by direct backpropagation.

**(2) Personalized model loss under samples class imbalanced.**

The severe imbalance in data categories significantly affects the performance of model training. To address the issue of class imbalance within the federated learning framework, this study adopts a personalized loss function based on margin correction and contrastive prediction encoding, guiding the training of imbalanced local models to approach the global balanced objective, thereby mitigating the performance degradation caused by class imbalance.

Typically, the prediction function consists of two parts: the feature representation learning function $f(\mathcal{x;}\theta_{r}):\mathcal{x}\to\mathcal{z}$ and the classifier function $g(\mathcal{z;}\theta_{o}):\mathcal{z}\to y$, where $\mathcal{x}$ is the input data, $\mathcal{z\in}{\mathbb{\mathbb{R}}}^{\boldsymbol{q}}$ is the feature representation of the original data with dimensionality $q$, and $y$ represents the final prediction output of the classifier. $\theta_{r}$is the parameter corresponding to the feature representation learning function, and $\theta_{o}$ is the parameter corresponding to the classifier function. Generally, $g(*)$ is a linear classification function, and the classification prediction score for the $c$-th class is as follows:

$\eta_{c}=g\left( \mathcal{z} \right)=\boldsymbol{W}_{c}\mathcal{z+}\boldsymbol{b}_{\boldsymbol{c}}$ （7）

where $\boldsymbol{W}_{c}$ and $\boldsymbol{b}_{\boldsymbol{c}}$ represent the weight and bias corresponding to the $c$-th class. Then, based on the softmax activation function, the predicted probability that the $i$-th training data $x_{i}$ is classified as class $y_{i}$ is computed as:

$p\left( y=y_{i} | x_{i};\theta_{r},\theta_{o} \right)=\frac{exp(\eta_{y_{i}})}{\sum_{c=1}^{C} exp(\eta_{c})}$ （8）

For a classification task with $C$ classes, the corresponding cross-entropy loss function is:

$\mathcal{l}\left( x_{i},y_{i};\theta_{r},\theta_{o} \right)=-log(\frac{exp(\eta_{y_{i}})}{\sum_{c=1}^{C} exp(\eta_{c})})$ （9）

As shown in **Figure S2**, for a standard binary classification task, the red represents the majority class, blue represents the minority class, and the hyperplane represents the classification hyperplane $H$. For the $c$-th class, the classification margin is calculated as:

$\begin{matrix} d_{c}=j\boldsymbol{W}_{\boldsymbol{c}}\left( \mathcal{z}_{\boldsymbol{1}}\boldsymbol{-}\mathcal{z}_{\boldsymbol{0}} \right)|| \\ =||\frac{\boldsymbol{W}_{\boldsymbol{c}}\left( \mathcal{z}_{\boldsymbol{1}}\boldsymbol{-}\mathcal{z}_{\boldsymbol{0}} \right)}{\boldsymbol{W}_{\boldsymbol{c}}\boldsymbol{\cdot}\boldsymbol{W}_{\boldsymbol{c}}}\boldsymbol{W}_{\boldsymbol{c}}|| \\ =\frac{\boldsymbol{W}_{\boldsymbol{c}}\left( \mathcal{z}_{\boldsymbol{1}}\boldsymbol{-}\mathcal{z}_{\boldsymbol{0}} \right)}{|\left| \boldsymbol{W}_{\boldsymbol{c}} \right||} \\ =\frac{\boldsymbol{W}_{\boldsymbol{c}}\mathcal{z}_{\boldsymbol{1}}\boldsymbol{+}\boldsymbol{b}_{\boldsymbol{c}}}{|\left| \boldsymbol{W}_{\boldsymbol{c}} \right||} \end{matrix}$ （10）

Here, $|\left| \boldsymbol{*} \right|\boldsymbol{|}$ represents the L2-norm. $\mathcal{z}_{\boldsymbol{1}}$ is an arbitrary data point in the feature space, and $\mathcal{z}_{\boldsymbol{0}}$ is a point on the classification hyperplane, satisfying $\boldsymbol{W}_{c}\mathcal{z}_{\boldsymbol{0}}\boldsymbol{+}b_{\boldsymbol{c}}\boldsymbol{=}0$. The term $proj\boldsymbol{W}_{\boldsymbol{c}}\left( \mathcal{z}_{\boldsymbol{1}}\boldsymbol{-}\mathcal{z}_{\boldsymbol{0}} \right)$ represents the distance from $\mathcal{z}_{\boldsymbol{1}}$ to the hyperplane $H$.


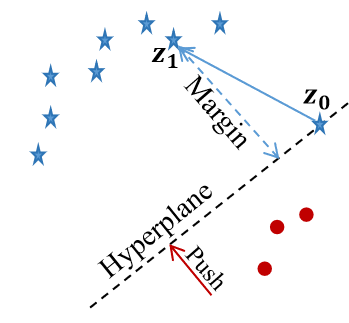


Figure S2 Schematic diagram of margin correction

Studies have shown that the decision boundary and prediction scores are correlated with the cardinality of each class, where the majority class tends to have a larger decision boundary and prediction scores [2]. Inspired by the above concept, this study adopts a margin calibration method (MARC) to calibrate the margin between positive and negative samples in medical images, aiming to achieve more balanced prediction scores. The adjusted margin distance is expressed as follows:

$\hat{d}_{c}$=${\omega_{c}\cdot d}_{c}+\beta_{c}$ （11）

Here, $\omega_{c}$and $\beta_{c}$ are margin calibration coefficients derived from the training samples, and $d_{c}$ is the margin after the standard training. By combining equations (7), (9), and (10), the margin-based cross-entropy loss function is formulated as follows:

$\mathcal{l}_{\mathrm{MARC}}\left( x_{i},y_{i};\theta_{r},\theta_{o} \right)=-log(\frac{exp(\omega_{y_{i}}\eta_{y_{i}+\beta_{y_{i}}||\boldsymbol{W}_{y_{i}}||})}{\sum_{c=1}^{C} exp(\omega_{c}\eta_{c+\beta_{c}||\boldsymbol{W}_{c}||})})$ （12）

In addition, the global model primarily focuses on aggregating low-frequency common information. Considering the significant differences in data distributions across different centers, to filter out task-relevant useful information and reduce noise and redundant information, and to further ensure that the aggregated parameters capture the shared invariant representations of all local models, this study extracts the fully connected layer parameters $f^{G}$ of the global model to represent global information. The fully connected layer parameters $f_{k}^{L}$ of the $k$-th local model are used to represent the information of the $k$-th local model. A similarity score based on the dot product operation is introduced, where a higher score indicates greater similarity between the two representations, and vice versa, a lower score indicates greater dissimilarity. The score is calculated as follows:

$s\left( x,y \right)=exp(\frac{x}{{||x||}_{2}}\cdot\frac{y}{{||y||}_{2}})$ （13）

Here, $\cdot$ denotes the dot product operation, and ${||*||}_{2}$​ represents the Euclidean norm.

The contrastive predictive coding (CPC) technique is adopted to align the distance between the global model representation and the local model representations, constructing a regularization loss term to constrain the model's learning process. The regularization loss is formulated as follows:

$\mathcal{l}_{\mathrm{CPC}}\left( f^{G},f^{L} \right)\mathbb{=-E[}log\frac{s(f^{G},f_{k}^{L})}{\sum_{i=1}^{K} s(f^{G},f_{i}^{L})}]$ （14）

Where $K$ is the number of clients and $\mathbb{E}$ denotes the expectation operator.

Finally, using the margin-calibrated cross-entropy loss function as the empirical loss and combining it with the contrastive predictive loss, the final structured loss function for the model is constructed as follows:

$\mathcal{l}oss$ = $\mathcal{l}_{\mathrm{MARC}}+\alpha*\mathcal{l}_{\mathrm{CPC}}$ （15）

Where $\alpha$ is the regularization parameter.

Based on the theory, a federated learning model is constructed to extract robust features.

**Supplementary S3: Feature extraction process**

To better utilize and understand the features learned by the model, this study used the convolutional kernels of each center's personalized model as feature extractors. Multiple feature maps were extracted from the local CT image data of each patient, and the average value of each feature map was calculated to create a joint radiomics feature. Given that the robust central model has a total of 7,616 convolutional kernels, a total of 7,616 federated radiomics features were extracted for each patient. These 7,616 features serve as the foundation for the model's assessment of early lung cancer recurrence, providing highly abstract representations. **Figure S3** illustrates the feature extraction process for patients.


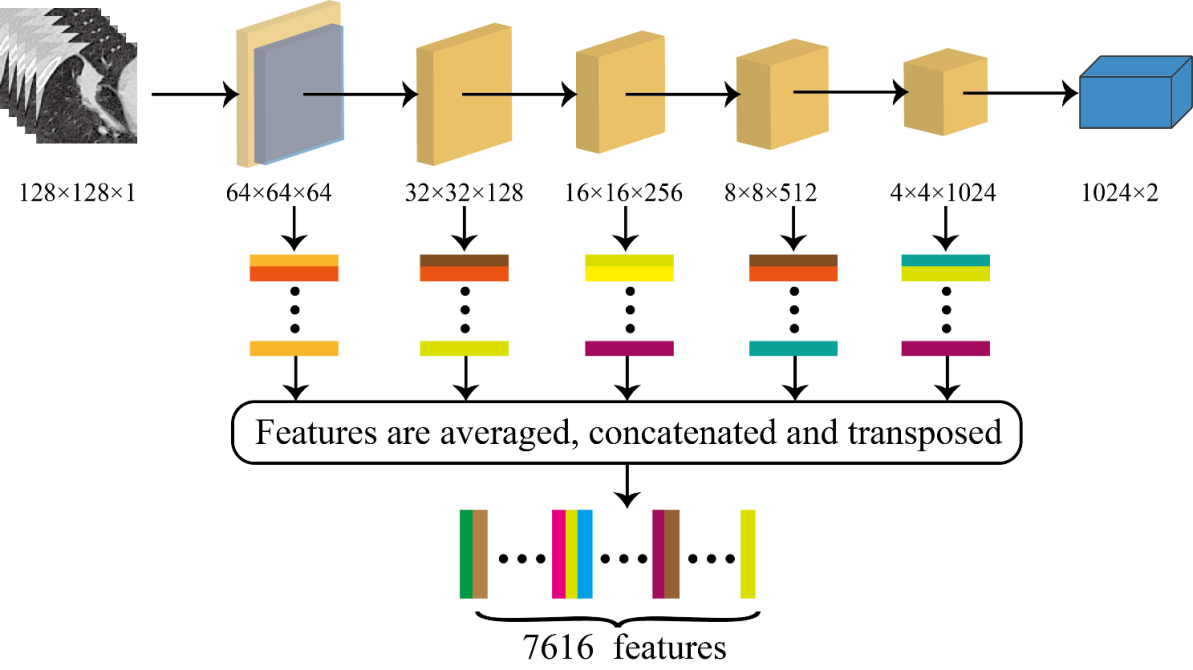


Figure S3 The feature extraction process

**Supplementary S4:** Ablation experiments of hyperparameters

To justify the selection of hyperparameters in the experiments, ablation experiments were performed in this study. Specifically, $r_{\max}$is the low-frequency threshold parameter with a range of 0 <$r_{\max}$< 0.5, and $\alpha$is the hyperparameter of the regularization term of the loss function with a range of $0<\alpha\leq1$.The specific ablation experiments are shown in Table S1 and S2.

Table S1 Analysis of hyper-parameter $r_{\max}$

| $r_{\max}$ | 0.1 | 0.2 | 0.3 | 0.4 |
| --- | --- | --- | --- | --- |
| Average AUC | 0.846 | 0.891 | 0.923 | 0.907 |

Table S2 Analysis of hyper-parameter $\alpha$

| $\alpha$ | 0.2 | 0.4 | 0.6 | 0.8 | 1.0 |
| --- | --- | --- | --- | --- | --- |
| Average AUC | 0.890 | 0.901 | 0.923 | 0.907 | 0.887 |

**Supplementary S5:** Results of Cox proportional risk regression analysis

Table S3 Results of Cox proportional risk regression analysis

| Center | Factor | Univariate Cox | | Multivariate Cox | |
| --- | --- | --- | --- | --- | --- |
|  |  | HR (95%CI) | P | HR (95%CI) | P |
| A | Age($\leq60 vs. >60$ years) | 0.236(0.079-0.708) | 0.010 | 0.093(0.019-0.453) | 0.003 |
|  | Gender(male $vs.$ female) | 0.610(0.234-1.587) | 0.311 | 1.211(0.432-3.452) | 0.706 |
|  | Longest diameter | 1.230(0.987-1.534) | 0.066 | 1.537(1.118-2.111) | 0.008 |
|  | CEA(normal $vs.$ elevated) | 0.404(0.165-0.988) | 0.047 | 1.365(0.529-3.520) | 0.520 |
|  | RFed | 4.597(2.899-7.291) | <0.001 | 5.454(3.009-9.883) | <0.001 |
| B | Age($\leq60 vs. >60$ years) | 0.841(0.449-1.575) | 0.589 | 1.088(0.564-2.099) | 0.802 |
|  | Gender(male $vs.$ female) | 0.822(0.439-1.539) | 0.540 | 1.136(0.596-2.167) | 0.698 |
|  | Longest diameter | 1.354(1.131-1.620) | 0.001 | 0.956(0.743-1.231) | 0.727 |
|  | CEA(normal $vs.$ elevated) | 0.370(0.197-0.698) | 0.002 | 0.538(0.280-1.037) | 0.064 |
|  | RFed | 4.732(3.283-6.819) | <0.001 | 4.749(3.210-7.027) | <0.001 |
| C | Age($\leq60 vs. >60$ years) | 1.436(0.456-4.526) | 0.536 | 3.701(0.841-16.293) | 0.084 |
|  | Gender(male $vs.$ female) | 0.810(0.257-2.551) | 0.718 | 1.140(0.334-3.886) | 0.835 |
|  | Longest diameter | 1.250(0.969-1.611) | 0.085 | 0.866(0.508-1.476) | 0.597 |
|  | CEA(normal $vs.$ elevated) | 1.113(0.301-4.112) | 0.872 | 4.905(0.979-24.579) | 0.053 |
|  | RFed | 3.541(2.200-5.700) | <0.001 | 6.744(2.847-15.978) | <0.001 |
| D | Age($\leq60 vs. >60$ years) | 1.131(0.283-4.523) | 0.862 | 1.229(0.125-12.084) | 0.860 |
|  | Gender(male $vs.$ female) | 0.855(0.204-3.579) | 0.831 | 21.339(0.782-582.433) | 0.070 |
|  | Longest diameter | 0.780(0.247-2.462) | 0.672 | 0.534(0.117-2.437) | 0.418 |
|  | CEA(normal $vs.$ elevated) | 1.059(0.214-5.248) | 0.944 | 1.532(0.158-14.874) | 0.713 |
|  | RFed | 4.034(2.189-7.435) | <0.001 | 9.176(2.835-29.693) | <0.001 |

Note: HR,hazard ratio; CEA, carcinoma embryonic antigen; CI, confidence interval; RFed, robust federated learning.

**Supplementary S6:** Results of calibration curve analysis

This study evaluated the calibration performance of RFed by plotting the calibration curve, as shown in **Figure S4**. The results show that the predicted probabilities are generally aligned with the actual observed probabilities, especially in the low-risk regions. In the middle-to-high probability ranges, the model tends to slightly overestimate the risk. The calibration slope is 0.892, which is close to the ideal value of 1, indicating that the predicted probabilities are reasonably well calibrated and trustworthy for downstream clinical interpretation. The slight overconfidence suggests room for further improvement using post-hoc calibration techniques such as temperature scaling, which we plan toexplore in future work.

**
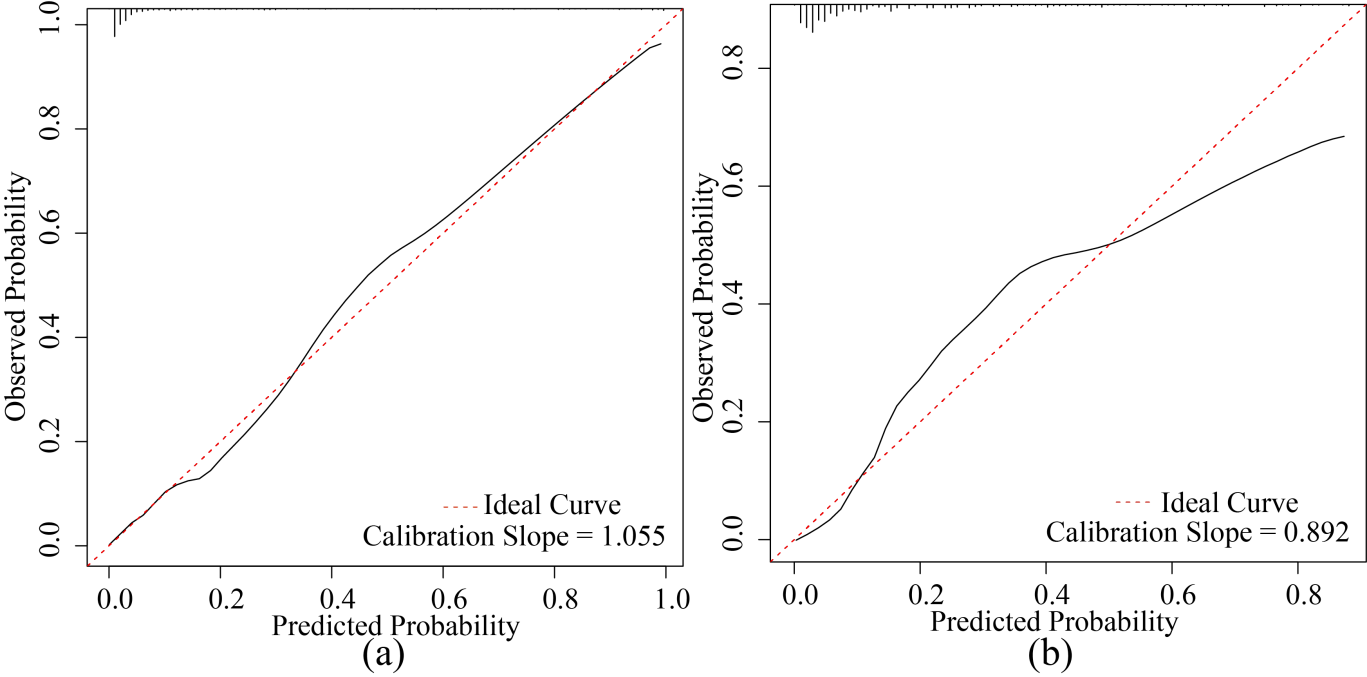
**

**Figure S4 C**alibration curves for the overall four-center. (a)Training set. (b) Test set

**Supplementary S7:** Correlation heat map of lung cancer non-progression group


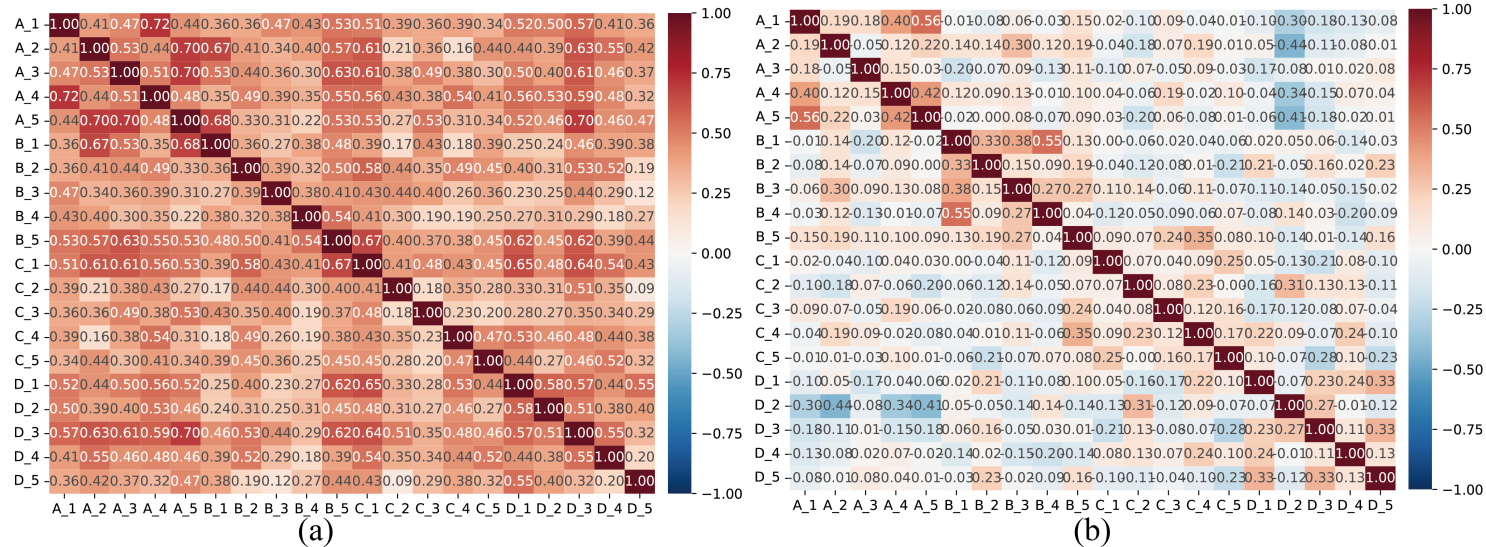


**Figure S5** Correlation heat map of center-common features and center-specific features. (a) Center-common feature of lung cancer non-progression group. (b) Center-specific features of lung cancer non-progression group. Notes: A_1, the first feature from center A

References:

1. Chen, R, Zhu, J, Hu, X, et al. Fault diagnosis method of rolling bearing based on multiple classifier ensemble of the weighted and balanced distribution adaptation under limited sample imbalance. ISA Transactions. 2021; 114 434-443. doi: 10.1016/j.isatra.2020.12.034
2. Wang Y, Zhang B, Hou W, et al. Margin Calibration for Long-Tailed Visual Recognition. Asian Conference on Machine Learning. 2022. <https://doi.org/10.48550/arXiv.2112.07225>
